# Exploring the Kamchatka Geothermal Region in the Context of Life’s Beginning

**DOI:** 10.3390/life9020041

**Published:** 2019-05-16

**Authors:** Vladimir N. Kompanichenko

**Affiliations:** Institute for Complex Analysis, 4 Sholom Aleyhem Street, Birobidzhan 679016, Russia; kompanv@yandex.ru; Tel.: +7-42622-24013

**Keywords:** origin of life, prebiotic analogues, hydrothermal systems, ionic and organic solutes

## Abstract

This article is a brief review of research in the Kamchatka geothermal region initiated by David Deamer and the author in 1999. Results obtained over the last 20 years are described, including a seminal experiment in which biologically important organic compounds were dispersed in a hot spring to determine their fate. Other investigations include ionic and organic composition of hydrothermal water, the source of hydrothermally generated oil, and pressure–temperature oscillations in hydrothermal systems. The relation of these results to research on the origin of life is discussed.

## 1. Introduction

**Figure 1:**
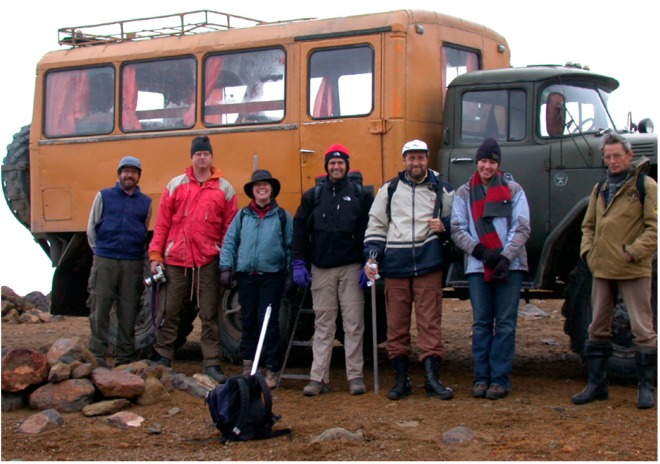
Participants on Mount Mutnovsky, September 2004.

About 20 years ago David Deamer and the author initiated an exploration of hydrothermal systems in the Kamchatka peninsula of Eastern Russia as a potential analogue of the prebiotic environment on early Earth. Our first discussion of possible fieldwork in Kamchatka was at the ISSOL conference in San Diego in 1999 and continued a year later during my visit to the University of California, Santa Cruz. We decided to organize two expeditions to Kamchatka hydrothermal sites in 2001 and 2004. I served as the local organizer for both, and for the 2001 trip David invited an American team of astrobiologists including Christopher McKay, Sherry Cady, John Spears, and Jonathan and Suzanne Trent. The second expedition in 2004 included Jamie Elsila and Meaghan Spencer, who were graduate students in Dick Zare’s lab at Stanford, two postdoctoral researchers from the Carnegie Institution of Washington, and our photographer, Tony Hoffman (Figure 1). Both expeditions were very successful for which we owe thanks to Gennady Karpov, Evgeny Vakin, and Georgy Yarotsky from the Institute of Volcanology and Seismology in Petropavlovsk.

During the second field expedition, David performed an experiment to be described later, which inspired much of his future research. We also collected water samples that were analyzed at the Institute for Volcanology and Seismology with the assistance of Gennady Karpov. Other sites sampled include the Uzon caldera, Valley of Geysers, and the Karymsky caldera lake.

Over the following ten years David and I stayed in close contact but worked in different geothermal systems. I continued exploration of the hydrothermal settings in Kamchatka together with my Russian colleagues―Valery Poturay, Gennady Karpov, Vladimir Rapoport, and Konstantin Shlufman―while David worked with his colleague Bruce Damer with field trips to Bumpass Hell on Mount Lassen in California, Yellowstone National Park and most recently New Zealand. A major goal of our research in Kamchatka has been to investigate organic matter in hot spring water and to characterize pressure–temperature oscillations in hydrothermal systems of deep boreholes. Recently David Deamer, Bruce Damer, and I integrated the relevant data obtained in Kamchatka and other geothermal regions, along with laboratory experiments, to discuss the connection between hydrothermal chemistry and the origin of cellular life [1]. I will now briefly describe the main results of my studies of Kamchatka hydrothermal sites.

## 2. General Description of the Kamchatka Geothermal Region

**Figure 2:**
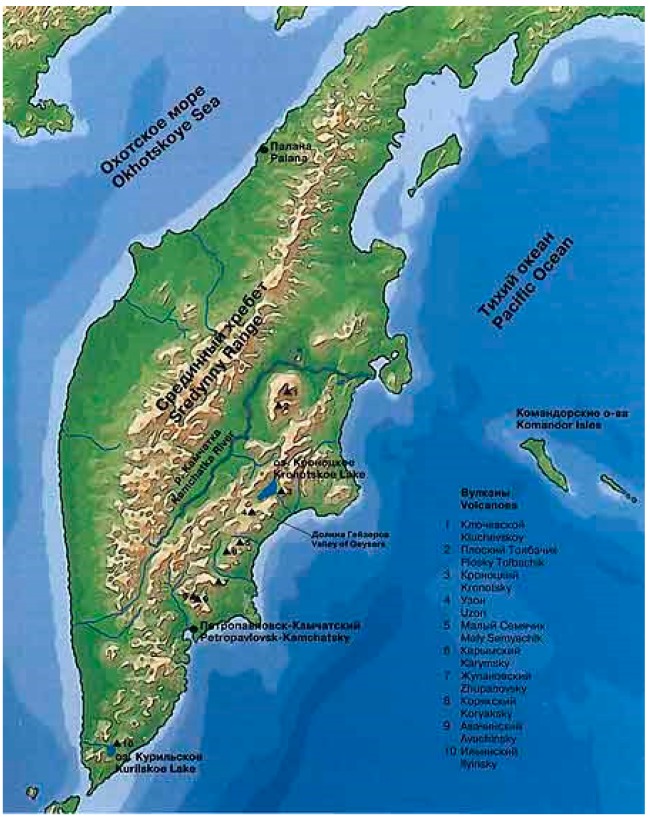
Map of the Kamchatka peninsula.

The Kamchatka peninsula is situated in northeastern Russia and spans a region of approximately 400 by 1200 km (Figure 2). The region contains 30 active volcanoes and approximately 270 hydrothermal fields. The most intensive hydrothermal activity traces a narrow zone along the axis of deep faults that cross the volcanic belts of eastern and central Kamchatka, with a breadth ranging from 30 to 50 kilometers. Over the years, hydrothermal processes in this area have attracted the attention of a number of Russian scientists [2,3,4,5,6].

Discharges of hydrothermal fluid in Kamchatka are very diverse, including boiling springs, geysers, fumaroles, and water vapor mixed with volcanic gases, such as sulfur dioxide. Seven regions of the Kamchatka peninsula have been explored: Mutnovsky, Uzon, Pauzhetsky, Valley of Geysers, Karymsky, Karymshinsky, and Paratunsky. The most substantial studies were carried out in the hydrothermal systems on Mount Mutnovsky and the Uzon caldera [2,3,4,5,6]. The water has pH values ranging from 1.5 to 10, and temperatures from 50 to 99 °C, while the temperature of the water-steam mixture from deep wells can be as high as 240 °C. Pressure may reach 40 bars at well-heads and 80 bars at 1 km depths.

## 3. The Fate of Organic Solutes in Hydrothermal Water

**Figure 3:**
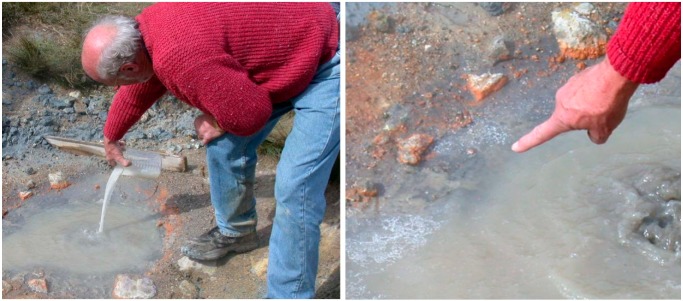
(Left) David Deamer added a “prebiotic soup” to a boiling pool in the Dachnoye thermal field on Mount Mutnovsky, and (right) points to the white froth of fatty acid membranes that immediately appeared around the edges. The pool was sampled over time and later analyzed. Surprisingly, all of the added solutes except the fatty acid were bound to the clay minerals, an observation that has implications for the fate of organic compounds in similar settings on the prebiotic Earth.

On 7 September 2004, a preliminary experiment was performed in which biologically important organic compounds were added to a natural hot pool (Figure 3). The experiment was inspired by Charles Darwin’s conjecture that life might have begun in a “warm little pond” so we jokingly referred to the pool as Darwin’s hot little puddle. The pool was approximately 0.7 × 0.7 meters with an estimated volume of 7 liters. The pH was 3.1 and the temperature at the boiling center was 97 °C. The pool was lined with a layer of grey clay mineral several centimeters deep. This was disturbed by the boiling which caused constant stirring and turbidity.

A mixture of the following compounds was added to the pool: 4 amino acids (glycine, l-alanine, l-aspartic acid, and l-valine, 1 gram each), four nucleobases (adenine, cytosine, guanine, and uracil, 1 gram each), sodium phosphate (3 grams), glycerol (2 grams), and myristic acid (1.5 grams). The goal of the experiment was to establish the fate of such compounds in natural hydrothermal conditions. Water samples (50 mL) and samples of the clay lining the pool were taken before addition of the powder, then at 1, 5, 30, 60, 120 min, 24 h, and 9 days later. Later analysis revealed that most of the added organics and phosphate were removed from solution with half times measured in minutes to a few hours. For instance, the two purines―guanine and adenine―disappeared from solution in about 2 h with a half time of 30 min. Even though cytosine was among the compounds added, it could not be detected in the analysis, but uracil was present at twice the expected concentration and disappeared with a half time of 2 h. The loss of cytosine was expected because at acidic pH ranges it readily undergoes deamination to uracil. When clay samples were analyzed, it was found that the organics could be released by addition of NaOH to bring the pH into the alkaline range, showing that with the exception of myristic acid, the organics had been adsorbed to the mineral surfaces at the acidic pH of the pool. This experiment was described in detail [7] and inspired David’s later work in other geothermal regions as well as laboratory simulations of hydrothermal environments. The ionic solutes in the pool are summarized in Table 1. The total concentration of ~2 mM is far below that of seawater, and therefore better able to support processes leading to the emergence of life.

## 4. Hydrochemistry of Hot Springs

Liquid water is one of the three widely accepted conditions for the origin of life together with a source of energy and organic compounds. Therefore, the ionic composition of hydrothermal water is exceptionally important. The hydrochemistry of hot spring water in Kamchatka has been extensively investigated by geologists from the Institute of Volcanology and Seismology in Petropavlovsk [2,3,4,5,6]. Thanks to their efforts, general chemical characteristics of hot spring water have been established. However, hydrothermal systems as potential analogues of the prebiotic environment on early Earth still require further exploration. Three aims have been achieved during our research.

**Figure 4:**
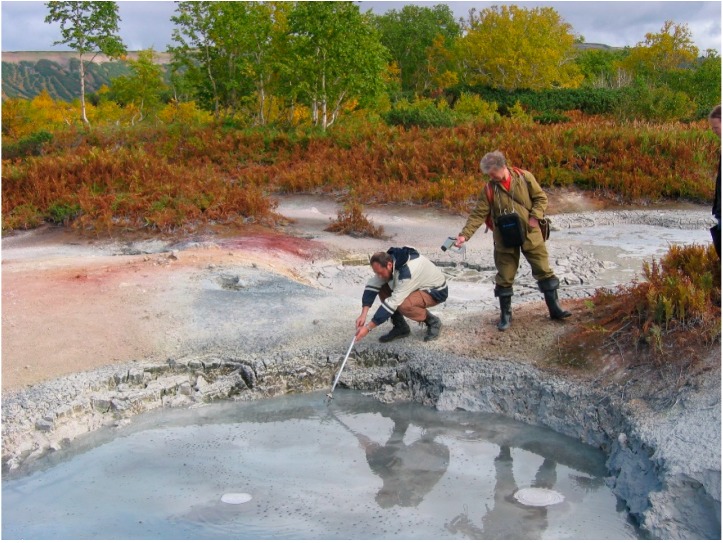
Hydrothermal pond in Uzon caldera. Vladimir Kompanichenko (on the left) takes a water sample while Gennady Karpov (on the right) remotely measures the temperature.

1. The hydrochemical zonality in the Uzon caldera was clarified. Previous researchers reported a change of water composition in the Uzon hydrothermal system from chloride-sodium in the center (just above the magmatic chamber) to sulfate in the intermediate zone and bicarbonate on the flank [3]. To establish this zonality, we took 20 samples of water from various fields in the Uzon caldera during the international expedition in 2004 (Figure 4). Among them, six samples belong to the chloride-sodium type, six to the sulfate type, and three to the bicarbonate type. Five analyses represent mixed chloride-sodium and sulfate types in which concentrations of Cl^−^ and SO_4_^2^^−^ are comparable. Table 2 summarizes typical analyses of each composition.

2. The general ratio between concentrations of univalent and divalent cations was characterized. The ratios Na^+^/K^+^ and Na+/Ca^2+^ are important for understanding the aqueous conditions related to the origin of life. For instance, Mulkidjanian et al. [9] considered the prevalence of K^+^ concentration over Na^+^ to be an important feature of aqueous prebiotic environment because K^+^ dominates the intracellular ionic concentration of cells today. However, in the Kamchatka geothermal region all of the samples analyzed sodium concentrations significantly exceeded those of potassium. This is also true for hot spring water in Yellowstone National Park. According to Milshteyn et al. [10], high concentrations of divalent ions such as Ca^2+^ inhibit self-assembly of lipid vesicles. The concentrations of Ca^2+^ in Kamchatka hot springs do not usually exceed 2–3 mM, a value that is conducive for self-assembly of lipid vesicles.

3. During our studies of organic solutes in Kamchatka thermal water, the dissolved organic compounds could be correlated with ionic composition. Such interdisciplinary approaches provided greater understanding of the complex geochemical characteristics of the selected field sites.

## 5. Organic Compounds in Hydrothermal Fluid

Previous investigations of dissolved organics in hydrothermal fluid in Kamchatka concerned volatile compounds [11] and nonvolatile amino acids [12]. However, a large group of moderately volatile organic compounds was virtually unexplored in 2005 when we obtained access to a Shimadzu GCMS-QP2010S spectrometer. The field sites in 11 hydrothermal systems in Kamchatka were sampled and analyzed, and the results were separated into two groups:

1. Water from hot springs and pools with a temperature of 55–98 °C inhabited by thermophiles and hyperthermophiles of archaea and bacteria.

2. These were compared to sterile condensates of water–steam mixtures from deep wells (600–2000 m) having temperatures between 108 °C and 175 °C.

We found that the sterile condensate of water–steam mixture contained 69 organic compounds that belong to 11 homologous series (Table 3). Organic compounds from the hot springs and pools supporting microbial populations are more diverse, containing 111 compounds in 14 homologous series (Table 3). Along with the analyses of volatile and nonvolatile organics, the total number of homologous series detected in the Kamchatka geothermal region is 24, including 243 distinct compounds. Several are prebiotically relevant, including nitrogen-containing compounds (amino acids, nitriles, amides, and nitrogen cycles) and lipid precursors (carboxylic acids, esters, alcohols, and aldehydes) [13,14,15].

The organic compounds associated with hydrothermal water have several potential sources. The most obvious is simple degradation of buried biological material, but abiotic synthesis is also a possibility [13]. Most of the compounds are derived from thermophilic microorganisms. A biological source was directly confirmed during the studies of amino acids in several thermal springs by Mukhin et al. [12] and oil droplets in Uzon caldera [16]. However, thermogenesis can also occur in local hydrothermal environments. We detected many moderately volatile compounds in the superheated sterile condensate of water–steam mixture from deep boreholes in Mutnovsky and Pauzhetsky hydrothermal systems [17]. Unlike Uzon caldera, the host rocks in Mutnovsky and Pauzhetsky areas are volcanic, not sedimentary, and do not contain substantial organic material, yet the condensate has short chain n-alkanes presumably generated by thermal geochemical processes. Glycine was also detected but at very low concentrations, less than a microgram per liter [12]. The occurrence of halogenated alkanes and aromatic hydrocarbons in the hot springs of the Mutnovsky volcano crater support abiotic thermogenesis because such compounds cannot be synthesized by thermophilic microorganisms.

## 6. Hydrothermally Generated Oil

Oil seeps in the central part of Uzon caldera were first observed by Russian scientists in the 1960s. The oils are present as water emulsions in porous rocks beneath the thin (5–8 cm) surface bed of clay, usually near hot mud pools and submerged griffons of thermal water. They can also be seen as an oil slick floating on the water surface. Thick layers of volcanic-sedimentary rocks have accumulated in the caldera’s lakes for the last 40,000 years and contain abundant detritus of higher plants and diatoms. Defining the process by which oil is generated takes into consideration an unusual combination of intense volcanic-hydrothermal processes and the host lacustrine basin with sediments rich in buried biological material.

The oil in Uzon caldera was first investigated in the 20th century [16], but the study carried out by Simoneit, Deamer, and the author provided significant new data [18]. Gas chromatography-mass spectrometry (GC-MS) was used to determine the primary constituents, and the 13C and 14C compositions provided information about the potential source and age of the oils. The 14C age was 1030 ± 40 yrs BP (measured) or 940 ± 40 yrs BP (conventional) which is the youngest hydrothermal petroleum reported to date. The delta 13C value is −30.6‰ versus the PDB standard. This is a clear indication that the oil was formed from biogenic detritus and not by abiotic synthesis from mantle carbon. The biogenic origin was confirmed by the presence of sterane and hopane biomarkers.

## 7. Pressure–Temperature Oscillations in Water–Steam Mixture in the Wells

**Figure 5:**
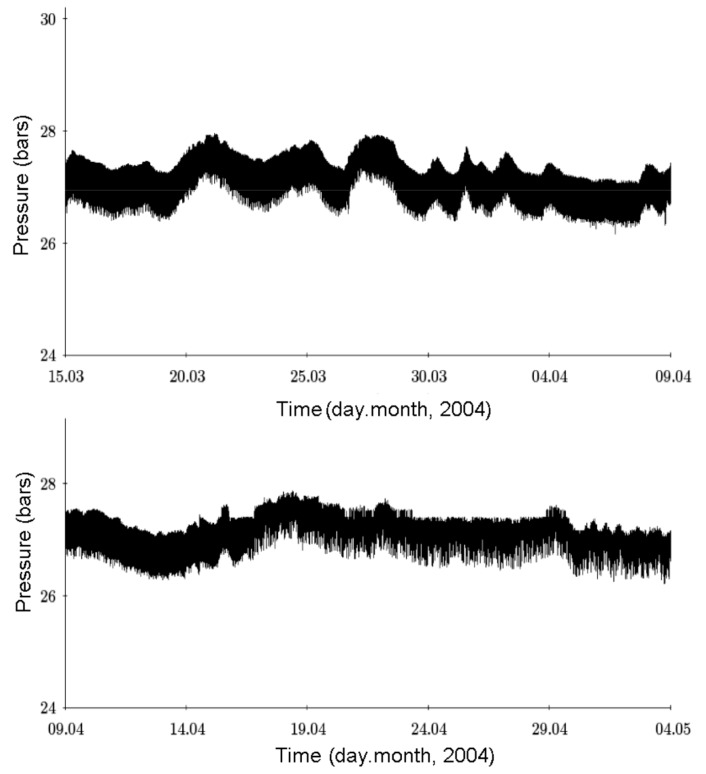
Pressure dynamics of water–steam mixture in well no. 30 in 2004. X-axis, day/month, Y-axis, pressure (bars).

The author has proposed that high-frequency oscillations of physicochemical parameters are required for the origin of life [8,19]. Such oscillations drive molecular synthesis and recombination and initiate a continuous evolutionary response of prebiotic microsystems to incessant stresses. In this context, several databases on pressure and temperature dynamics in the wells in Mutnovsky hydrothermal system were mathematically processed [20]. The monitoring of the wells was carried out both at the surface and at depth. The minimal interval between the measurements was a few minutes, and two portions of the pressure dynamics over a 25-day period in well no. 30 are shown on Figure 5.

Short-term variability of pressure at a depth of 1 km consists of two main components: Regular micro-oscillations having periods of 10–20 min and amplitudes up to 1 bar, and irregular macrofluctuations with much longer pressure variations measured in days. The pressure varied between 24.9 and 29.6 bars. We also observed a third component of the pressure dynamics characterized by sudden pressure changes and high frequency fluctuations with periods of less than 5 min. The correlation coefficient between pressure and temperature was estimated from measurements at the wellheads and was highly positive, ranging from 0.89 to 0.99.

## 8. Current Research

All life requires liquid water, and presumably life began in liquid water. This means that the ionic composition of hydrothermal water is significant if we are to understand the origin of life. The reason is that the major compounds associated with life processes are typically ionic solutes having carboxylate, phosphate, and amine groups, and the chemical properties of these groups are strongly affected by the hydrogen ion and divalent cation concentrations. An obvious example is availability of phosphate, which is essential to life. At alkaline pH ranges of 8 and above, and in the presence of calcium, mineral apatite precipitates and removes phosphate from solution, but at acidic pH ranges phosphate is soluble even in the presence of calcium. Another example is the inhibitory effect of divalent cations on the ability of long chain monocarboxylic acids to assemble into membranes.

The results of the Kamchatka exploration represent a good basis for further interdisciplinary research relevant to life’s beginnings, and particularly the role of fluctuating environments [8,19]. The author has proposed that oscillating conditions are required for life to emerge, as well as availability of organic matter, an aqueous medium, and a source of chemical energy. A key idea is that self-sustaining life processes were launched by continuous response of prebiotic microsystems to incessant physicochemical oscillations in the environment. Our observations of pressure–temperature oscillations and wet–dry cycles in the hydrothermal systems of Kamchatka have inspired laboratory experiments on prebiotic chemistry [21,22,23].

Because most of Earth’s liquid water is in the ocean, it has been generally assumed that life began in the ocean, perhaps at hydrothermal vents. However, there is reason to believe that thermodynamic hurdles and divalent cation concentrations inhibit self-assembly of membranes and polymerization reactions required for life to begin [24]. The results of laboratory simulations and fieldwork performed in Kamchatka, Yellowstone Park, and New Zealand suggest that fresh water distilled from seawater and provided as precipitation to volcanic land masses and hot springs may be a more conducive medium for the origin of life.

## Figures and Tables

**Table 1:** Ionic composition and concentrations in the boiling pool shown in Figure 3.

Cations	NH_4_^+^	Na^+^	Ca^2+^	Fe^3+^	K^+^	Mg^2+^	Al^3+^	H^+^
mM	0.9	0.6	0.31	0.11	0.12	0.13	0.11	1.0
Anions	SO_4_^2^^−^	Cl^−^	SiO_4_					
mM	2.2	0.04	3.1					

**Table 2:** Characteristic ionic solutes in the Uzon hydrothermal system (adapt from [8]).

Water Type	Chloride-Sodium Type			Sulfate Type			Bicarbonate Type		
Components	mg/L	mg-eq/L	% mg-eq/L	mg/L	mg-eq/L	% mg-eq/L	mg/L	mg-eq/L	% mg-eq/L
pH	6.76			4.56			6.51		
HCO_3_^−^	12	0.2	0.3	< 0.6			654.1	10.72	70.7
Cl^−^	2234	63	96	2.1	0.06	12.8	29.1	0.82	5.4
SO_4_^2-^	96	2	3.1	19.2	0.4	85.1	172.9	3.6	23.8
F^-^	1.2	0.06	0.1	0.2	0.01	2.1	0.2	0.01	0.1
**Sum_an_**	2247.4	65.26	100	21.5	0.47	100	856.3	15.15	100
Na^+^	1307.4	56.87	89.5	6.4	0.28	56	164.7	7.16	45.9
K^+^	139.3	3.56	5.6	<0.39			11.6	0.3	1.9
NH_4_^+^	3	0.17	0.3	0.1			< 0.1		
Ca^2+^	52.9	2.64	4.2	3.2	0.16	32	92.2	4.6	29.5
Mg^2+^	2.9	0.24	0.4	0.7	0.06	12	43.2	3.55	22.7
Fe^2+^	< 0.3			< 0.3					
Fe^3+^_com_	< 0.3			< 0.3					
H				0.1	0.07	0.8			
**Sum_cat_**	1505.5	6348	100	10.4	0.5	100	311.7	15.61	100
H_3_BO_3_	18.5			1.2			6.2		
H_4_SiO_4sol_	190			48			182.6		
H_4_SiO_4col_	452			77			11.3		
Salinity	4413.4			158.4			1368.1		

Analyses were made at the Analytical Center of the Institute of Volcanology and Seismology in Petropavlovsk by V.S. Sergeeva.

**Table 3:** Homologous series of moderately volatile organic compounds detected in the hot springs supporting microbial life (first number) and sterile condensate of water–steam mixtures from wells (second number) (adapt from [8]).

Homologous Series	Compounds in Springs/in Wells
Alkanes	21/21
Isoalkanes	17/7
Isoprenes	0/2
Cycloalkanes (naphthenes)	2/0
Alkenes	7/0
Aromatic hydrocarbons	13/26
Halogenated aromatic hydrocarbons	9/2
Alcohols	5/4
Aldehydes	4/2
Ketones	3/2
Carboxylic acid	14/1
Esters	11/0
Steroids	3/0
Terpenes	1/1
Lactams	1/0
Sulfur-containing hydrocarbons	0/1
